# Structure refinement and anisotropic atomic displacement parameters of 1M Illite: Rietveld and pair distribution function analysis using synchrotron X-ray radiation

**DOI:** 10.1107/S1600576725004170

**Published:** 2025-06-20

**Authors:** Seungyeol Lee

**Affiliations:** a Department of Earth and Environmental Sciences Chungbuk National University 28644Cheongju-si Republic of Korea; University of Silesia in Katowice, Poland

**Keywords:** illite, synchrotron X-ray radiation, Rietveld refinement, pair distribution function analysis, anisotropic atomic displacement parameters

## Abstract

Illite, a widespread clay mineral, plays a pivotal role in geological processes, notably as an indicator in diagenetic and hydro­thermal alteration environments, and possesses significant industrial relevance in applications including ceramics, construction and catalysis. However, challenges including its nanoscale crystallinity, structural disorder and frequent interstratification with other clay minerals have hindered detailed structural characterization using conventional X-ray diffraction (XRD) techniques. This study employs integrated synchrotron XRD and pair distribution function (PDF) analysis to elucidate the crystal structure of the 1M illite polytype, yielding the first determination of its anisotropic atomic displacement parameters (*U*_aniso_). These *U*_aniso_ parameters provide critical insights into atomic dynamics and static disorder within the structure, enabling a more refined understanding of structure–property relationships. This integrated approach, combining synchrotron XRD, Rietveld refinement and PDF analysis, yields a comprehensive structural characterization, capturing both average crystallographic and local atomic arrangements. Considering illite’s widespread geological occurrence and industrial importance, this high-precision structural dataset, especially the determined *U*_aniso_ values, provides a crucial benchmark for future modeling and simulation efforts targeting accurate prediction of its physicochemical behavior.

## Abstract 2

Combined synchrotron X-ray diffraction and pair distribution function analyses have been used to determine, for the first time, the anisotropic atomic displacement parameters of illite, offering new insights into its atomic dynamics.

## 1. Introduction

Illite is a non-expansible, dioctahedral clay mineral belonging to the mica group, distinguished by its deficiency of interlayer cations – primarily K^+^ – compared with true micas. It is widely distributed in soils, sediments and sedimentary rocks, and exhibits distinctive physicochemical properties such as high specific surface area, plasticity, low permeability and thermal stability (Pevear, 1999 ▸; Rieder *et al.*, 1998 ▸; Gaudette *et al.*, 1964 ▸; Harvey & Murray, 1997 ▸; Chistidis, 2010 ▸). These properties make illite a valuable indicator of geological processes, including weathering, diagenesis, pedogenesis and hydro­thermal alteration (Gaudette *et al.*, 1964 ▸). Its structural changes under varying pressure and temperature conditions provide valuable information for reconstructing the thermal history and burial depth of sedimentary basins, while its influence on porosity and permeability significantly impacts hydro­carbon reservoir quality (Pevear, 1999 ▸).

Beyond its geological relevance, illite has a broad range of industrial and environmental applications. Its characteristic surface area and capacity facilitate the adsorption of environmental pollutants, including heavy metals and radionuclides (Harvey & Murray, 1997 ▸; Christidis, 2010 ▸). Its plasticity, low permeability and thermal stability make it a key component in ceramic products and construction materials such as bricks and tiles (Meunier & Velde, 2013 ▸). In addition, its surface acidity and surface properties render it suitable for catalytic applications in chemical and petrochemical industries (Gaudette *et al.*, 1964 ▸; Christidis, 2010 ▸). Despite its widespread occurrence and multifunctional utility, a detailed understanding of illite’s atomic structure remains elusive. This is largely due to its inherently fine-grained, disordered nature and frequent intergrowth with other clay minerals, such as smectite (Drits *et al.*, 1996 ▸; Gualtieri *et al.*, 2008 ▸; Brigatti, 2013 ▸).

The development of modern synchrotron radiation sources and high-resolution detectors has enabled atomic-scale structural studies that were previously inaccessible (Masadeh, 2016 ▸; Lee & Xu, 2020 ▸). High-energy synchrotron X-ray diffraction (XRD) provides the broad-*Q*-range data required for accurate atomic pair distribution function (PDF) analysis (Masadeh, 2016 ▸; Lee & Xu, 2020 ▸; Lee *et al.*, 2021 ▸; Lee & Xu, 2019 ▸; Billinge & Levin, 2007 ▸). In particular, the anisotropic atomic displacement parameters (*U*_aniso_) which describe direction-dependent atomic vibrations and static disorders offer deeper insight into atomic-scale dynamics than their isotropic counterparts (*U*_iso_) (Lee & Xu, 2020 ▸; Lee *et al.*, 2021 ▸). Although *U*_aniso_ parameters are traditionally derived from single-crystal XRD, the small crystal size of typical illite samples often precludes this approach (Lee *et al.*, 2021 ▸; Lee & Xu, 2019 ▸). Conventional powder XRD combined with Rietveld refinement yields reliable average structural information, including *U*_iso_, but remains insufficient for detailed modeling of anisotropic atomic displacement parameters (Lee & Xu, 2020 ▸; Lee & Xu, 2019 ▸).

PDF analysis, based on total scattering data, complements conventional diffraction methods by capturing both Bragg and diffuse scattering. This technique enables simultaneous investigation of local, intermediate and long-range atomic arrangements, making it particularly well suited for disordered, nanocrystalline or heterogeneous materials such as illite (Lee & Xu, 2020 ▸; Billinge & Levin, 2007 ▸; Toby & Egami, 1992 ▸). Using real-space refinement programs such as *PDFgui* or *RMCProfile*, PDF analysis can extract detailed structural parameters, including * U*_aniso_, with high sensitivity to local environments (Billinge & Levin, 2007 ▸; Farrow *et al.*, 2007 ▸; Tucker *et al.*, 2007 ▸).

Given that illite generally occurs as small, disordered and intergrown nanocrystals, structural analysis via conventional single-crystal or neutron diffraction is impractical. To overcome these challenges, this study adopts a synergistic approach that combines high-resolution synchrotron XRD with PDF analysis. While synchrotron XRD provides precise information on the average long-range structure, PDF analysis excels in resolving local structural features, including the determination of *U*_aniso_ parameters (Lee & Xu, 2020 ▸; Lee *et al.*, 2021 ▸; Lee & Xu, 2019 ▸). Accordingly, the primary aim of this study is to report, for the first time, the *U*_aniso_ values for the 1M polytype of illite. These parameters offer a refined view of direction-dependent atomic displacements and static disorder, thereby advancing our understanding of the structure–property relationships that govern illite’s diverse behaviors.

Illite structures are known to exhibit varying degrees of rotational disorder, with stacking sequences involving 0°, ±60° or ±120° rotations between adjacent layers. This variation distinguishes ordered polytypes such as 1M (0°) and 2M_1_ (±120°) from the more common, disordered 1M_d_ varieties found in natural samples. In this context, the present study focuses on the structurally ordered 1M illite polytype and employs a combined Rietveld and PDF analysis approach, based on synchrotron XRD data, to comprehensively characterize its atomic structure.

## 2. Materials and methods

The Silverton illite sample used in this study was obtained from the S. W. Bailey collection in the Department of Geoscience at the University of Wisconsin–Madison. The sample originates from the Silverton caldera, situated between the towns of Silverton and Ouray, Colorado. This tertiary-aged caldera, part of the San Juan volcanic field, comprises Oligocene andesitic and rhyodacitic lava flows and tuffs of the Silverton volcanic group (Eberl *et al.*, 1987 ▸). Illite appears to have formed via hydro­thermal alteration of fault gouge within fractures. The <2 µm size fraction was separated from the bulk sample using standard sedimentation techniques (Jackson, 2005 ▸). The isolated fine fraction was chemically treated using a sodium acetate buffer to remove carbonates, followed by sodium di­thio­nite to eliminate iron oxides.

The treated <2 µm illite fraction was analyzed using a JEOL JSM-IT510 scanning electron microscope at the Department of Earth and Environmental Sciences, Chungbuk National University. The scanning electron microscope was operated at an accelerating voltage of 15 kV with a working distance of 10 mm. Prior to imaging, the sample was sputter-coated with a thin layer of platinum to enhance conductivity and improve image quality. Elemental analysis was conducted using a Rigaku ZSX Primus II X-ray fluorescence (XRF) spectrometer at the university’s Research Support Center. The instrument utilized a rhodium target X-ray tube operated at 60 kV and 40 mA. The treated sample was prepared as a pressed powder pellet to ensure homogeneity and quantitative accuracy. All measurements were performed under vacuum conditions to minimize air attenuation and enhance the detection of light elements.

Synchrotron-based XRD experiments were performed at beamline 17-BM of the Advanced Photon Source (APS), Argonne National Laboratory, using monochromatic X-rays with a wavelength of 0.24152 Å. The treated <2 µm sample was packed into polyimide tubes with an inner diameter of 1 mm. Calibration of the sample-to-detector distance and beam center was carried out using a LaB_6_ standard. Diffraction data from an empty polyimide tube were also collected for background subtraction. Crystal structure refinement was conducted using the *TOPAS 5* software (Bruker, MA, USA; Coelho, 2018 ▸). An initial structural model based on known illite structures was employed, with chemical composition obtained from scanning electron microscopy (SEM) and XRF analyses. Peak profiles were modeled using a pseudo-Voigt function, and refinement parameters included unit-cell dimensions, fractional atomic coordinates and isotropic atomic displacement parameters (*U*_iso_).

PDF measurements were conducted at the same beamline (17-BM) of the APS using the same X-ray wavelength (0.24152 Å). The prepared sample was loaded into a 1 mm diameter polyimide tube. Two-dimensional diffraction patterns were recorded in transmission geometry using an amorphous silicon area detector. Each exposure was set to 1 s and repeated 300 times, yielding a total acquisition time of 300 s. Calibration of the detector distance and beam center was again performed using a LaB_6_ standard, and background scattering was measured from an empty tube under identical conditions. Two-dimensional diffraction images were integrated into one-dimensional intensity-versus-*Q* profiles using the *GSAS-II* software (Toby & Von Dreele, 2013 ▸). The resulting data, collected up to a maximum momentum transfer (*Q*_max_) of 19.6 Å^−1^, were transformed into real-space PDFs using *PDFgetX3* (Juhás *et al.*, 2013 ▸). The instrumental resolution parameters were set to *Q*_damp_ = 0.045 Å^−1^ and *Q*_broad_ = 0.069 Å^−1^. Subsequent fitting and structural refinement of the PDF data were performed using *PDFGui* (Farrow *et al.*, 2007 ▸).

## 3. Results and discussion

### 3.1. Microstructural characteristics and chemical composition

**Figure 1:**
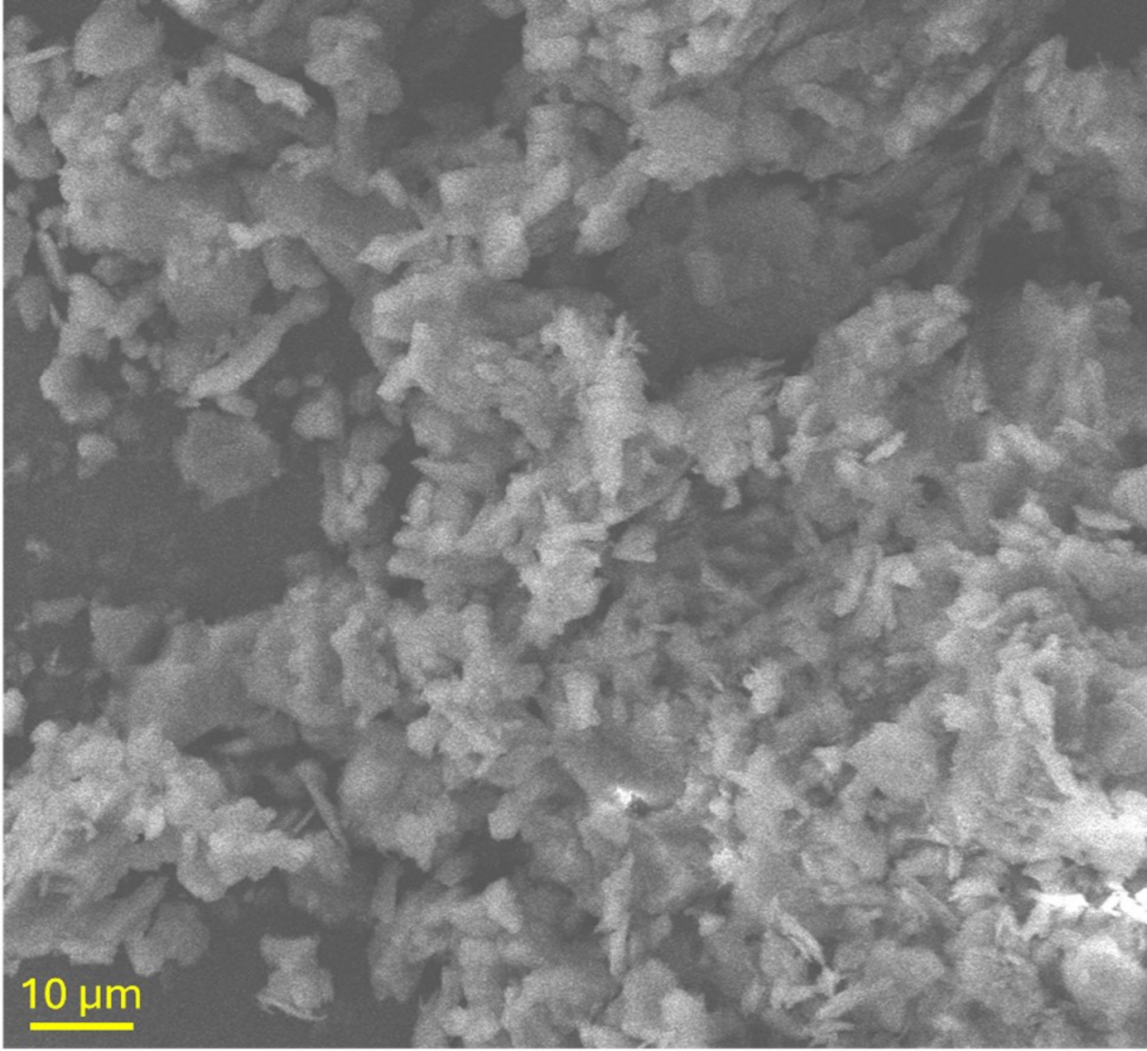
SEM image of the studied illite particles, revealing their typically small size (often less than 10 µm) and characteristic plate- or flake-like morphology.

SEM revealed that the studied illite particles are generally fine grained, with most measuring less than 2 µm in size. They display a characteristic plate- or flake-like morphology typical of phyllosilicate minerals (Fig. 1 ▸). These plate-like crystals frequently exhibit irregular edges and are commonly observed as stacked aggregates. In addition to the dominant plate-like forms, occasional fibrous or lath-like morphologies were also observed, which may have resulted from hydro­thermal alteration of precursor minerals (Drits *et al.*, 1996 ▸).

The chemical composition of the treated illite sample, as determined by XRF analysis, is as follows: 48.92 wt% SiO_2_, 33.04 wt% Al_2_O_3_, 1.38 wt% MgO, 0.05 wt% CaO, 0.10 wt% Na_2_O and 9.33 wt% K_2_O. Fe_2_O_3_ was below the detection limit. The relatively high Al_2_O_3_ and low MgO contents indicate an aluminium-rich and magnesium-poor composition. The significant K_2_O content is consistent with the typical chemical signature of illitic clays, in which potassium ions occupy interlayer sites to compensate for negative structural charge (Gualtieri *et al.*, 2008 ▸).

### 3.2. Structural analysis using synchrotron X-ray diffraction

**Figure 2:**
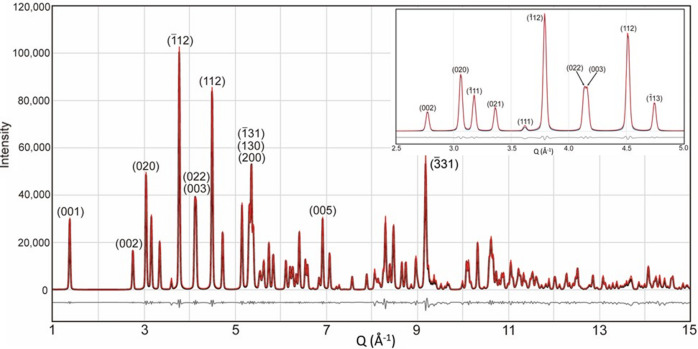
Synchrotron XRD pattern of the studied illite. Experimental data are shown in black, and the calculated pattern from Rietveld refinement is shown in red. The gray line below the patterns represents the residual difference between the experimental and calculated data. The inset in the upper right corner shows a magnified view of the 2.5–5 *Q* region. Indices (*hkl*) corresponding to the 1M_tv_ illite structure are indicated. The goodness-of-fit is 7.15%.

Synchrotron XRD analysis provided critical insights into the crystallographic structure of the illite sample. Prior to analysis, the <2 µm fraction was confirmed to be mineralogically pure and predominantly composed of illite, as determined within the XRD detection limits. As shown in Fig. 2 ▸, the diffraction pattern is marked by a distinct series of basal 00*l* reflections, which confirm the layered structure characteristic of phyllosilicate minerals. The basal spacing (*d*_001_), calculated from the 001 reflection, was 10.224 Å. Notably, the broadening of diffraction peaks – especially in higher-order 00*l* reflections – indicates considerable stacking disorder, commonly observed in hydro­thermally altered illite (Meunier & Velde, 2013 ▸).

A more detailed analysis of the diffraction pattern enabled identification of the dominant polytype. The reflections were indexed to a 1M_tv_ illite structure, a disordered monoclinic polytype characterized by a one-layer (1M) stacking sequence and *trans*-vacant (tv) octahedral layers. This polytype is distinct from the well ordered 2M_1_ variety, which typically displays sharp and intense 02*l*, 11*l* and 13*l* reflections in the corresponding region (Fig. 2 ▸). The weak to moderate intensities of these reflections in our data support the predominance of the 1M_tv_ polytype (Dong & Peacor, 1996 ▸).

To obtain detailed structural parameters, Rietveld refinement was performed using the synchrotron XRD data (Fig. 2 ▸). The refinement confirmed a monoclinic structure (space group *C*2/*m*), consistent with a *trans*-vacant configuration in the octahedral sheet. The refined unit-cell parameters are *a* = 5.2028 (3) Å, *b* = 8.9803 (5) Å, *c* = 10.2241 (7) Å and β = 101.549 (11)°. The site occupancies were in good agreement with the chemical composition derived from XRF analysis. The refinement yielded a weighted profile *R* factor (*R*_wp_) of 7.15%, indicating a satisfactory fit between observed and calculated patterns. The full set of refined parameters, including atomic coordinates and site occupancies, is presented in Table 1 ▸.

These unit-cell parameters were compared with those of a well characterized 1M_tv_ illite from a similar geological setting (Table 2 ▸). The *a* and *b* parameters exhibited close agreement, suggesting similar octahedral sheet dimensions and chemical composition. However, the average tetrahedral T—O bond length in the present sample was slightly larger (1.6431 Å) than that of the reference sample (1.6387 Å) (Drits *et al.*, 2010 ▸). This difference is consistent with partial Al^3+^ substitution for Si^4+^ in the tetrahedral sheet, as the larger ionic radius of Al^3+^ typically results in increased T—O distances. Alternatively, variations in tetrahedral rotation may also contribute to the observed differences (Gualtieri *et al.*, 2008 ▸).

### 3.3. Structural analysis using pair distribution function analysis

**Figure 3:**
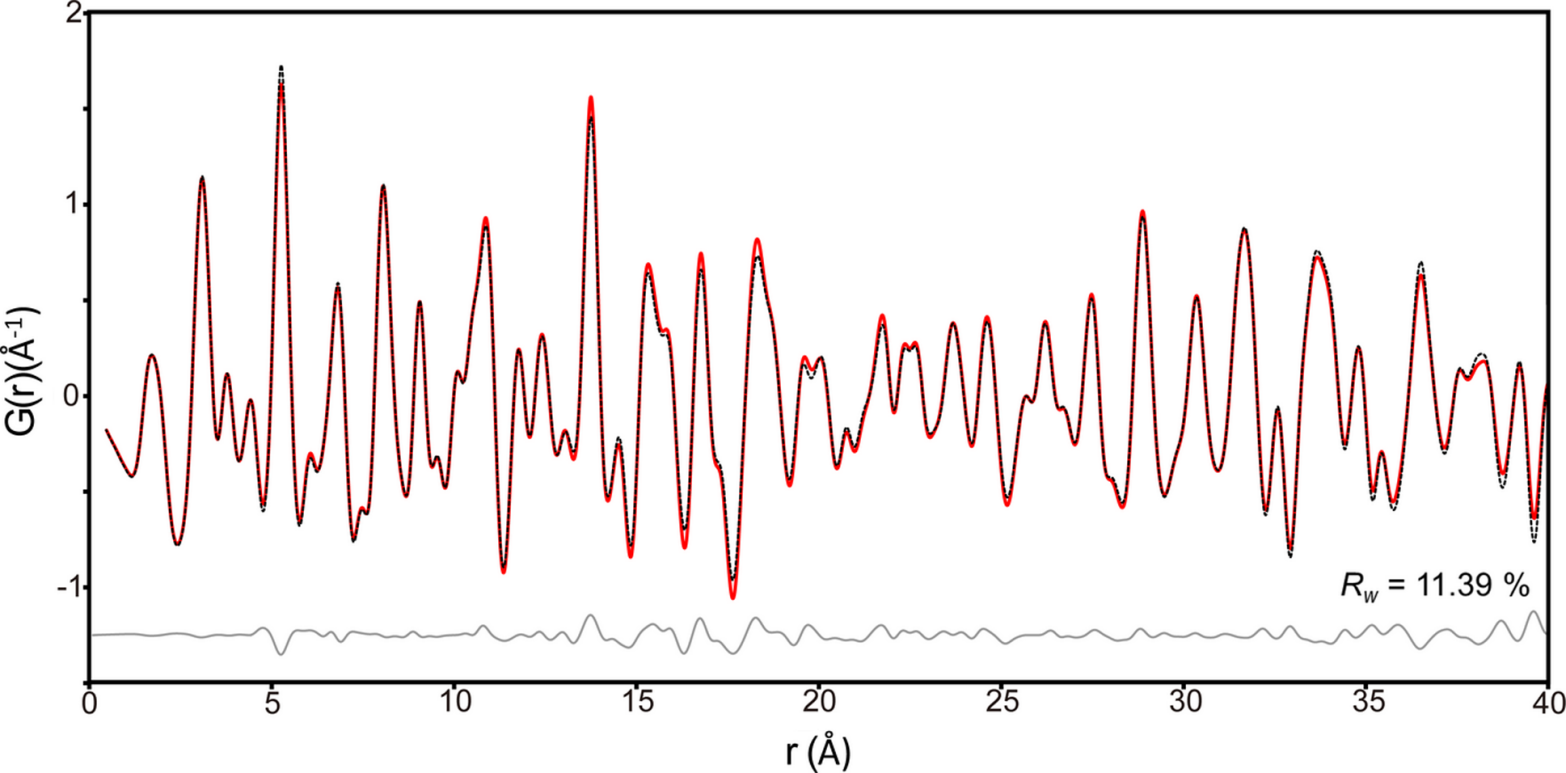
Synchrotron X-ray PDF analysis of illite. The experimental PDF, derived from X-ray total scattering data and covering the range 1 to 40 Å, is shown as a black dashed line. The calculated PDF is shown by the red line, and the difference between the experimental and calculated patterns is shown in gray. The goodness-of-fit (*R*_w_) is 11.39%.

The experimental X-ray PDF, *G*(*r*), of the 1M_tv_ illite was obtained over the *r*-range 1 to 40 Å and is shown in Fig. 3 ▸. The first prominent peak, located near 1.74 Å, corresponds to overlapping contributions from Si—O, Al—O and Al—OH atomic correlations within the tetrahedral and octahedral sheets. Specifically, this peak reflects the average Si—O bond length of 1.64 Å in SiO_4_ tetrahedra, the Al—O bond length of approximately 1.93 Å in AlO_6_ octahedra and the Al—OH distance near 1.91 Å.

PDF refinement was conducted using the structural model derived from the Rietveld refinement (Fig. 2 ▸, Table 1 ▸) as the starting configuration. During refinement, atomic coordinates, occupancy and unit-cell parameters were fixed, and the anisotropic atomic displacement parameters (*U*_aniso_) were refined for each atom. Although the initial PDF refinement included atomic coordinates, occupancy and unit-cell parameters, the resulting values showed significant distortion. Consequently, we directly used the average structure values obtained from Rietveld refinement. This methodology mirrors previous studies on materials like kaolinite, moganite and cristobalite (Lee & Xu, 2020 ▸; Lee *et al.*, 2021 ▸; Lee & Xu, 2019 ▸). The refinement yielded good agreement between the experimental and calculated PDFs, with a residual R_w_ value of 11.39% (Fig. 3 ▸). The refined *U*_aniso_ values and other parameters are summarized in Table 1 ▸.

Although the peak positions in the experimental and calculated PDFs match well, minor discrepancies in peak intensities are evident (Fig. 3 ▸). These deviations can be attributed to several factors. First, the structural model assumes perfect crystallographic periodicity and does not fully account for the stacking disorder inherent in 1M_tv_ illite. Second, PDF analysis is more sensitive to atomic thermal vibrations than conventional Rietveld refinement, which may explain the observed variations in intensity. Third, while Rietveld analysis provides an average long-range structural model, PDF analysis probes local atomic environments. Therefore, the observed mismatches may reflect subtle local structural deviations, such as those arising from the *trans*-vacancy configuration or minor structural defects and impurities.

**Figure 4:**
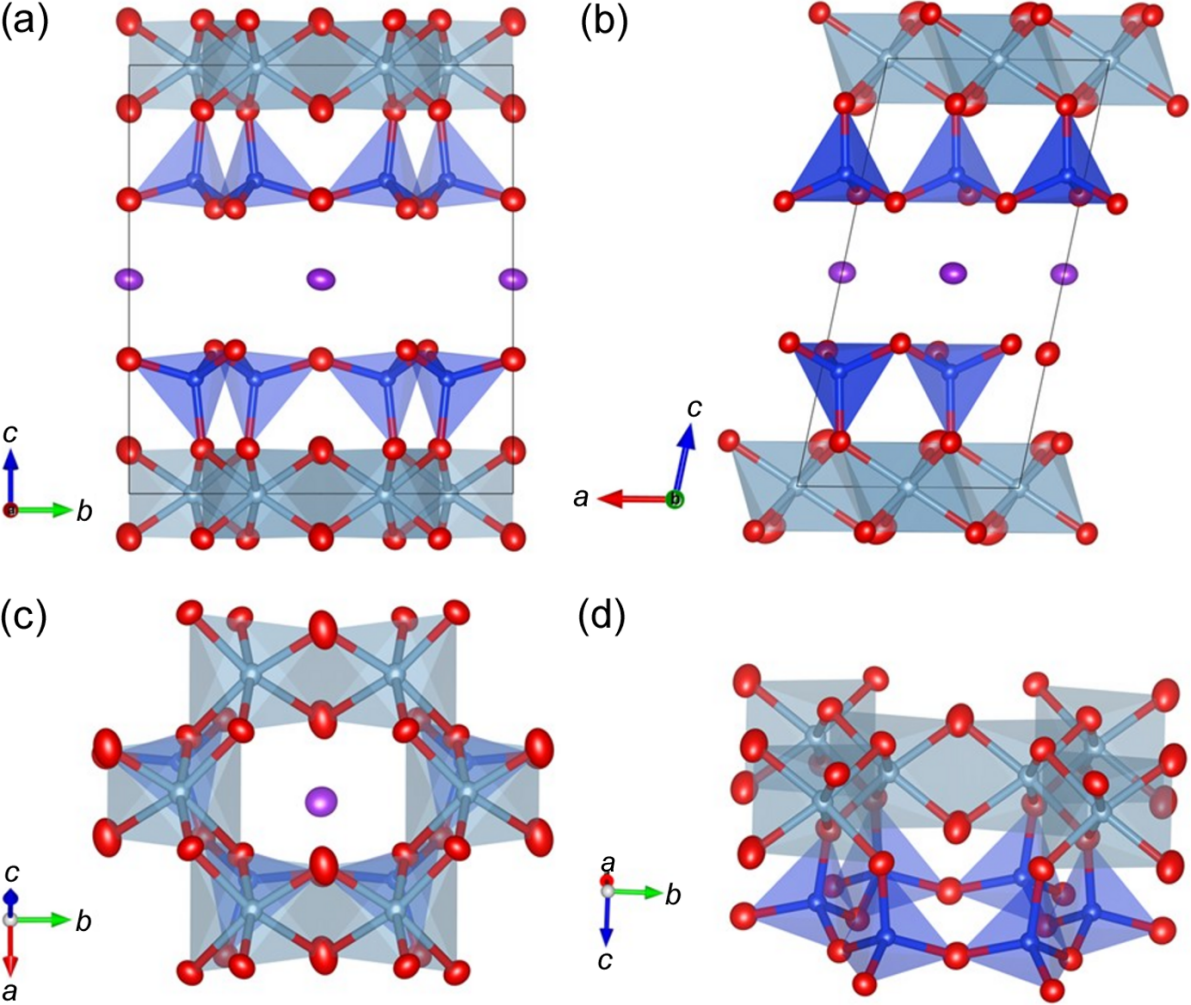
Structural models of illite derived from Rietveld refinement and PDF analyses. Displacement ellipsoids, representing 80% probability, are shown for each atom. Silicon atoms are depicted in blue, oxygen in red, aluminium in light blue and potassium in purple.

Notably, the PDF analysis enabled the refinement of *U*_aniso_, providing new insights into the directional nature of atomic vibrations (Table 1 ▸). Previous studies have demonstrated the superiority of the PDF method in revealing local structural details compared with conventional crystallographic techniques (Lee & Xu, 2019 ▸; Billing & Levin, 2007 ▸; Masadeh *et al.*, 2022 ▸; Lee *et al.*, 2022 ▸). This study is the first to report *U*_aniso_ parameters for a natural 1M_tv_ illite. The refined structural model, including anisotropic displacement ellipsoids, is illustrated in Fig. 4 ▸. The illite structure is composed of six-membered rings of corner-sharing SiO_4_ tetrahedra that form tetrahedral sheets, which are linked by layers of edge-sharing AlO_6_ octahedra. The ellipsoids associated with oxygen atoms in the tetrahedral sheet are elongated perpendicular to the Si–Si vector, suggesting a precession-like vibrational motion. The hydroxyl (OH) groups exhibit notably larger anisotropic displacements than other oxygen atoms (Table 1 ▸), likely due to their structural role in accommodating the *trans*-vacancy configuration and balancing local charge, similar to their function in kaolinite (Lee & Xu, 2020 ▸).

In addition, the *U*_aniso_ values for the interlayer cation site, predominantly occupied by K^+^, were larger than those observed for the tetrahedral and octahedral framework sites. This trend is consistent with previous findings by Gualtieri (2000 ▸), who reported elevated *U*_iso_ values for the interlayer site using XRD-based Rietveld refinement. The agreement between our PDF-derived *U*_aniso_ parameters and prior crystallographic data supports the robustness and reliability of our refinement results, as presented in Table 1 ▸.

## 4. Conclusions

This study employed an integrated analytical approach – combining SEM, XRF, high-resolution synchrotron XRD, Rietveld refinement and PDF analysis – to achieve a comprehensive structural characterization of the 1M polytype of Silverton illite. The results confirm its Al-rich, Mg-poor composition, pronounced stacking disorder and the dominance of a *trans*-vacant configuration within the octahedral sheets. The refined unit-cell parameters and atomic coordinates are consistent with previously reported data (Drits *et al.*, 2010 ▸). Furthermore, the use of synchrotron radiation significantly improved the precision of structural measurements. Notably, this approach enabled the first reliable determination of anisotropic atomic displacement parameters (*U*_aniso_) for a natural 1M_tv_ illite. These parameters provide critical insights into the directionality of atomic vibrations and local structural dynamics within the phyllosilicate framework. The refined structural model, particularly the detailed *U*_aniso_ values, contributes substantially to our understanding of illite’s structure–property relationships. Given illite’s widespread occurrence and industrial relevance, the high-precision structural dataset presented here offers a valuable foundation for future modeling and simulation efforts aimed at predicting its physicochemical behavior with greater accuracy.

## Supplementary Material

Crystal structure: contains datablock(s) I. DOI: 10.1107/S1600576725004170/jur5001sup1.cif

Supporting figure and table. DOI: 10.1107/S1600576725004170/jur5001sup2.pdf

CCDC reference: 2449650

## Figures and Tables

**Table 1:** The structural parameters of illite were determined from synchrotron XRD and scattering data as follows: unit-cell parameters, atomic coordinates and site occupancy were derived from Rietveld refinement, and anisotropic atomic displacement parameters (*U*_aniso_) were calculated using PDF analysis T: tetrahedral, O: octahedral, Int: interlayer.

Atom site	Occ.	*x*	*y*	*z*	*U*_11_	*U*_22_	*U*_33_	*U*_12_	*U*_13_	*U*_23_
Si_T_	0.81	0.4192 (4)	0.3281 (3)	0.2684 (3)	0.0115 (7)	0.0124 (9)	0.0120 (9)	−0.0008 (1)	0.0012 (2)	−0.0011 (1)
Al_T_	0.19									
Al_O_	0.94	0.5	0.1667	0	0.0155 (9)	0.0148 (9)	0.0161 (10)	−0.0004 (1)	0.0016 (3)	0.0011 (2)
Mg_O_	0.06									
K_Int_	0.84	0	0.5	0.5	0.0288 (15)	0.0315 (17)	0.0187 (11)	−0.0021 (3)	0.0040 (5)	−0.0018 (3)
Ca_Int_	0.03									
Na_Int_	0.01									
O1	1	0.3493 (4)	0.3091 (4)	0.1051 (3)	0.0177 (9)	0.0215 (11)	0.0195 (9)	0.0027 (4)	0.0024 (4)	−0.0018 (3)
O2	1	0.4987 (5)	0.5	0.3135 (3)	0.0217 (8)	0.0244 (8)	0.0230 (7)	0.0007 (5)	0.0013 (6)	−0.0008 (4)
O3	1	0.6711 (6)	0.2241 (5)	0.3352 (5)	0.0166 (6)	0.0193 (7)	0.0178 (7)	−0.0018 (6)	0.0021 (5)	−0.0011 (3)
OH	1	0.4187 (5)	0	0.1011 (2)	0.0370 (11)	0.0250 (10)	0.0313 (11)	0.0033 (7)	−0.0031 (5)	−0.0021 (5)

**Table 2:** Unit-cell parameters and bond distances of the refined illite, calculated from Fig. 2 ▸, compared with those reported by Drits *et al.* (2010 ▸) (all values considered in this comparison were obtained from the refinement of XRD patterns)

	This study	Drits *et al.* (2010 ▸)
*a*	5.2028 (4)	5.2021 (4)
*b*	8.9803 (5)	8.9797 (6)
*c*	10.2240 (7)	10.226 (8)
β	101.548 (11)	101.57 (1)
Si—O1	1.645 (5)	1.6363 (13)
Si—O2	1.640 (3)	1.63888 (13)
Si—O3	1.645 (5)	1.63946 (19)
Si—O3	1.645 (5)	1.6402 (4)
Average	1.6431	1.6387
Al—O1	1.929 (3)	1.9300 (4)
Al—O1	1.929 (3)	1.9300 (4)
Al—O1	1.934 (4)	1.9494 (6)
Al—O1	1.934 (4)	1.9494 (6)
Al—OH	1.9140 (15)	1.9105 (5)
Al—OH	1.9140 (15)	1.9105 (5)
Average	1.9260	1.9300
K—O2 (×2)	2.906 (3)	2.9088 (8)
K—O3 (×4)	2.878 (5)	2.8835 (9)
Average	2.8874	2.8919
