# Supplementary material for: Structure refinement and anisotropic atomic displacement parameters of 1M Illite: Rietveld and pair distribution function analysis using synchrotron X-ray radiation
Source: J Appl Crystallogr. 2025 Jun 20;58(Pt 4):1185–90. doi: 10.1107/S1600576725004170 (PMC12321013; doi:10.1107/S1600576725004170)
Supplement: Supplementary file 2 [file j-58-01185-sup2.pdf]

JOURNAL OF
APPLIED
CRYSTALLOGRAPHY

Volume 58 (2025)

Supporting information for article:

Structure refinement and anisotropic atomic displacement parameters of 1M Illite: Rietveld and PDF analysis using Synchrotron X-ray radiation

Seungyeol Lee

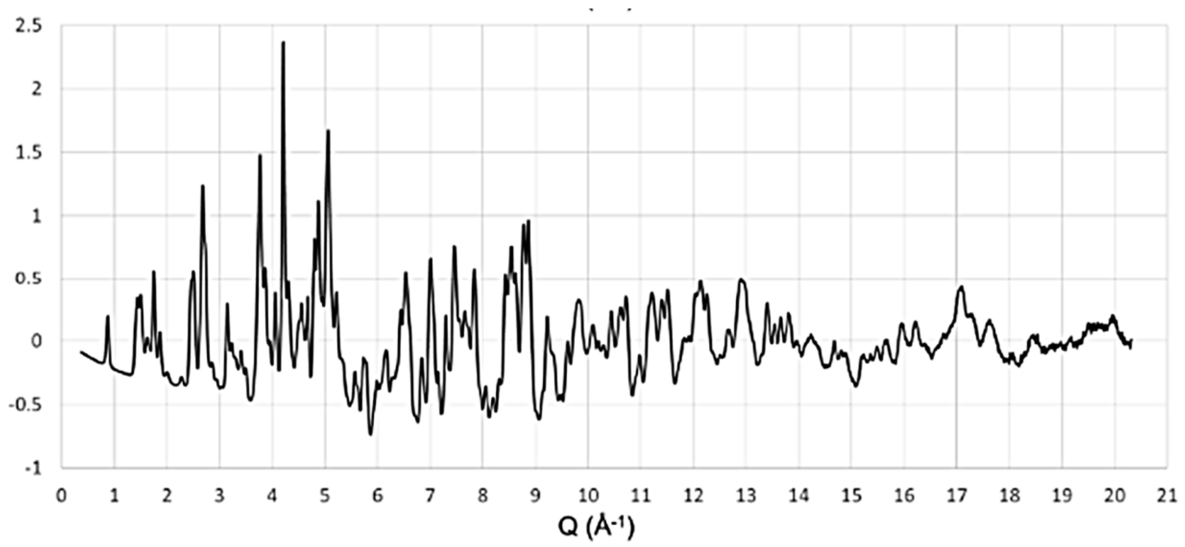

Figure S1. X-ray PDF analysis of reduced structure function, F(Q), data for illite from the Silverton caldera, Colorado, USA.

Table S1. The structural parameters of illite were determined by Rietveld refinement of synchrotron X-ray diffraction data.
(T: tetrahedral, O: octahedral, Int: interlayer)

Atom Site	Occ.	x	y	z	U_{iso}
Si_T	0.81	0.4192(4)	0.3281(3)	0.2684(3)	0.01205(2)
Al_T	0.19				
Al_O	0.94	0.5	0.1667	0	0.01658(3)
Mg_O	0.06				
K_{Int}	0.84	0	0.5	0.5	0.02744(5)
Ca_{Int}	0.02				
Na_{Int}	0.01				
O1	1	0.3493(4)	0.3091(4)	0.1051(3)	0.01975(4)
O2	1	0.4987(5)	0.5	0.3135(3)	0.02347(5)
O3	1	0.6711(6)	0.2241(5)	0.3352(5)	0.01866(3)
OH	1	0.4187(5)	0	0.1011(2)	0.03348(7)
